# Sulphite addition during steam pretreatment enhanced both enzyme-mediated cellulose hydrolysis and ethanol production

**DOI:** 10.1186/s40643-022-00556-w

**Published:** 2022-06-29

**Authors:** Na Zhong, Richard Chandra, Minna Yamamoto, Timo Leskinen, Tom Granström, Jack Saddler

**Affiliations:** 1 Department of Wood Science, Faculty of Forestry, Forest Products Biotechnology and Bioenergy Group, The University of British Columbia, 2424 Main Mall, Vancouver, BC Canada; 2 Trinity Western University, 22500 University Dr, Langley, BC Canada; 3 St1 Oy, Firdonkatu 2, Helsinki, Finland; 4 VTT Technical Research Centre of Finland Ltd., 02044 Espoo, Finland

**Keywords:** Sulphite steam pretreatment, Acid steam pretreatment, High consistency hydrolysis, Fermentation, Detoxification, Whole slurry

## Abstract

Sulphite addition during steam pretreatment of softwoods under acidic, neutral and alkaline conditions was assessed to try to minimize lignin condensation. Although pretreatment under neutral/alkaline conditions resulted in effective lignin sulphonation, non-uniform size reduction was observed. In contrast, acidic sulphite steam treatment at 210 °C for 10 min resulted in homogenous particle size reduction and water-insoluble component that was 62% carbohydrate and 33% lignin. This carbohydrate-rich substrate was readily hydrolyzed and fermented which indicated the lack of fermentation inhibitors in the steam-pretreated whole slurry. The use of high solid loading (25% w/v) resulted in a hydrolysis yield of 58% at an enzyme loading of 40 mg protein/g glucan and efficient fermentation (46.6 g/L of ethanol). This indicated that the addition of acidic sulphite at the steam pretreatment of softwoods improved both the enzymatic hydrolysis and fermentation of steam-pretreated whole slurries.

**Graphical Abstract:**

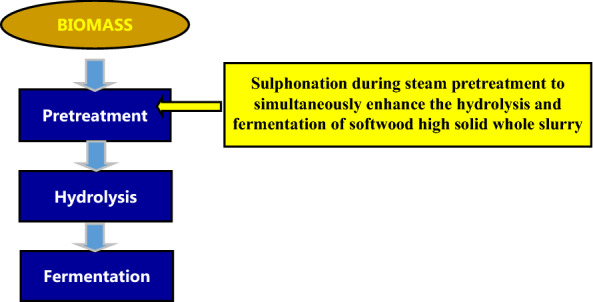

**Supplementary Information:**

The online version contains supplementary material available at 10.1186/s40643-022-00556-w.

## Introduction

Acid-catalyzed steam pretreatment of softwood can result in the homogenous size reduction of the water-insoluble fraction (WIF) while solubilizing hemicellulose into a water-soluble fraction (WSF). This increased the enzyme-mediated hydrolysis of the cellulose component while increasing the fermentability of the wood-derived sugars (Chandra et al. 2009). However, the dissolution of hemicellulose also results in the enrichment of the lignin component in the water-insoluble fraction. The high lignin content of water-insoluble, cellulose-rich fraction has been shown to impede the enzymatic hydrolysis of the cellulose (Zhong 2018; Ji et al. 2022). The lignin in the pretreated softwood is also prone to acid-induced lignin condensation which increases the non-productive binding between lignin and cellulase enzymes during enzymatic hydrolysis (Rio et al. 2011; Chandra et al. 2016; Kumar and Sharma 2017). As a result, high enzyme loadings are typically required to achieve acceptable enzymatic hydrolysis yields (Baig 2020). In more recent work, sulphonation post-treatments have been successfully used to modify the lignin, consequently increasing enzyme-mediated cellulose hydrolysis (Kumar et al. 2012).

However, as mentioned earlier, the acidic conditions used during steam pretreatment typically solubilize most of the hemicellulose-derived hexose sugars. Thus, to try to achieve higher sugar and ethanol yields it would be preferable if the water-soluble and water-insoluble fractions could be combined. Although the solubilization of hemicellulose component has been shown to facilitate cellulose hydrolysis, by increasing enzyme accessibility, the acidic conditions (pH < 2) and the high temperatures typically required to contend with the high recalcitrance of softwoods during steam pretreatment results in the production of both sugar and lignin-derived fermentation inhibitors (e.g., furans, aliphatic acids and phenolic compounds) (Jönsson et al. 2013; Cavka et al. 2011; Palmqvist and Hahn-Hägerdal 2000).

Sulphite has also been shown to detoxify the water-soluble substrate fraction of steam-pretreated substrates resulting in increased fermentation yields (Jönsson et al. 2013; Soudham et al. 2011). To try to overcome the challenges associated with both the hydrolysis and fermentation of the whole slurry (i.e., the water-soluble plus the water-insoluble fractions), previous work assessed the effectiveness of sulphite post-treatments to simultaneously sulphonate lignin-associated with the cellulose-rich water-insoluble component, to enhance enzymatic hydrolysis, while detoxifying the water-insoluble component (Zhong et al. 2019). However, it was apparent that the condensed nature of the lignin resulting from steam treatment limited the sulphonation of the water-insoluble fraction. The sulphite post-treatment of the steam-pretreated softwood whole slurry (combined water-soluble and water-insoluble fractions) was also carried out at 70 °C, to limit the thermal degradation of the solubilized sugars. However, increasing the hydrolysis yield from 56 to 68% at the lower temperature required the use of longer residence times (12 h) and the addition of 2% sodium carbonate to the 8% sulphite in order to raise the pH and to improve the reactivity of the lignin with sulphite. However, it was likely that the lignin would be more reactive if the sulphite was added at higher temperatures prior to the lignin condensation that occurs during the steam pretreatment process (Li et al. 2007; Lin 2016; Nakagame et al. 2011). In this work, we assessed the efficacy of sulphonating wood chips during steam pretreatment under neutral/alkaline or acidic conditions to maximize lignin modification with the goal of enhancing both the enzymatic hydrolysis and fermentation of the high solids concentration whole slurry.

## Material and methods

### Biomass

The wood chips used in this study were Mountain beetle killed lodgepole pine wood chips that were kindly provided by Canfor. Inc, British Columbia, Canada. From the Klason analysis described in “Analytical methods” section below, the composition of the starting lodgepole pine biomass was determined to be 1.9% arabinan, 3.1% galactan, 42% glucan, 5.8% xylan, 12% mannan and 29% lignin.

### SO_2_-catalyzed steam pretreatment

The size of lodgepole pine wood chips were screened and retained between 6 and 38 mm. Wood chips were impregnated with SO_2_ 12 h prior to pretreatment. Based on our previous work, half of the SO_2_ initially added to the biomass will be retained after SO_2_ impregnation. Therefore, to obtain 4% (wt/wt of the dry substrate) SO_2_ for pretreatment, 8% SO_2_ (wt/wt of the dry substrate) was added to 200 g (oven dry basis) wood chips and kept at room temperature for 12 h. The amount of SO_2_ adsorption was determined by difference in the total weight of the wood chips before and after the addition of the sulphur dioxide gas. Steam pretreatment was conducted at 200 °C for 5 min in a 2-L StakeTech II batch steam gun (constructed by Stake Tech, Norval, Ontario, Canada). From the results of Klason analysis (described in “Analytical methods” section below), the SO_2_-catalyzed steam-pretreated lodgepole pine contained 48% glucan, 45% lignin, 3.7% mannan, 1.3% xylan, 0.1% arabinan, 0% galactan.

### Sulphite steam pretreatment

Prior to pretreatment, 200 g of dry lodgepole pine chips were mixed in a plastic bag with water containing either sodium sulphite or sodium sulphite plus sodium carbonate. The pretreatment was performed under alkaline conditions at a solid:liquid ratio of 2:1. The wood chips mixed with the chemical solution were subsequently heated at 70 °C in a water bath for 12 h. Another set of experiments using acidic bisulphite prior to steam pretreatment also used the same protocol. In each case, the wood chips with chemical solution which had been impregnated at 70 °C were steam pretreated in the steam gun, at 160 °C for 10 or 20 min. The chips and the liquor resulting from the steam pretreatment were then separated by vacuum filtration. In the case of the pretreatment performed using two stages, the chips that were initially subjected to the pretreatment at 160 °C were then subjected to a second stage steam pretreatment at the specified temperatures and residence times. The mechanical size reduction of the sulphite steam-pretreated substrates was performed by subjecting the substrates to lab-scale twin-gear refiner (Super Angel 8500).

### Hydrolysis

This study utilized the Cellic Ctec3 enzyme preparation which was kindly provided by Novozymes (Franklinton, NC). The protein concentration of Cellic Ctec3 was 227 mg/mL as measured using the ninhydrin protein assay (Mok et al. 2015). The enzyme activity of the enzyme was 171.7 FPU/mL (using Whatman No. 1 filter paper as a substrate for the filter paper assay). The hydrolysis of the steam-pretreated lodgepole pine substrate was conducted at pH 4.8 and 50 °C in a rotary shaker at 150 rpm for 48 h. High solids-loading whole slurry enzymatic hydrolysis was conducted in duplicate at 25% (w/v) consistency. The whole slurry contained 25% (w/v) water-insoluble and 75% (w/v) water-soluble fractions. Prior to the enzymatic hydrolysis, the whole slurry was adjusted to a pH of 4.8 using NH_4_OH. The 25% (w/v) consistency hydrolysis was conducted at 50 °C in flasks that were incubated in an orbital shaker running at 150 rpm (Thermo Electron Model 480). The hydrolysis yields (%) of substrates were calculated based on the amount of glucose released in the hydrolysate divided by the theoretical cellulose content of the substrates.

### Fermentation

Fermentation was conducted at a working volume of 20 mL in 50-mL septa bottles (Millipore Sigma Canada) with butyl-PFTE seals. The *Saccharomyces cerevisiae* T_2_ strain that was employed in this research was kindly provided by Tembec Limited (Temiscaming, Quebec, Canada). This T_2_ strain has been widely used in the sulphite mill for the fermentation of the softwood-derived hemicellulose hydrolysate (Kapu et al. 2013). Prior to fermentation, the T_2_ strain was cultivated in YPD media at 30 °C in an orbital shaker running at 150 rpm (Thermo Electron Model 480) for propagation. NH_4_OH was used to adjust the pH of either the lodgepole pine hydrolysate or dissolving pulp hydrolysate to 5.5 prior to yeast inoculation. The cultivated T_2_ strain was inoculated in fermentation bottles containing 20 ml of either the lodgepole pine whole slurry hydrolysate or dissolving pulp hydrolysate and was incubated at 30 °C in an orbital shaker running at 150 rpm (Thermo Electron Model 480). During fermentation, 400-μL samples were taken at various time intervals. The samples were centrifuged at 5000 rpm for 5 min and the supernatant was stored at − 80 °C prior to further analysis.

### Analytical methods

The chemical composition of the substrates was determined by the method described by Sluiter et al. (2006). the fiber quality analyzer is used to measure the fiber length, width and size distribution of the substrates. Dried biomass samples were mounted on aluminum SEM stubs using double-sided tape and sputter-coated with 10 nm Au/Pd (80:20 mix) then imaged on a Hitachi S-2600 VP-SEM (Tokyo, Japan). The monomeric sugars concentration was determined by HPLC analysis, as previously described by Bura et al. (2009). The acid groups content of the pretreated substrates and biomass were measured using the conductometric method according to Katz et al. (1984). The fiber length and fiber width of the substrates were determined using a high-resolution fiber quality analyzer (FQA) (LDA02; Op Test Equipment, Inc., Hawkesbury, ON, Canada) in accordance with the procedure described by Robertson et al. (1999) Scanning electron microscopy (SEM) images were taken by a JEOL JSM-5600 SEM (Japan) with the freeze-dried sample. Prior to imaging, the sample was sputter-coated with a Pd–Au alloy to build up the charge on the surface.

Gas chromatography equipped with a HP-Innowax column (15 m × 0.53 mm) was used to test the ethanol concentration. Helium was utilized as the carrier gas and the flow rate of carrier gas is 20 mL/min. The temperatures of the injection unit and flame ionization detector (FID) were set at 175 and 250 °C, respectively. Prior to the test, the oven was heated up to 45 °C and kept for 2.5 min. Then temperature was raised to 110 °C at a rate of 20 °C/min and later held at 110 °C for 2 min. Internal standard for ethanol testing were prepared by using ethanol (Sigma) and butanol (0.5 g/L) (Fisher).

The fermentation/hydrolysis inhibitors including furfural, 5-hydroxymethylfurfural (HMF) as well as acetic acid were analyzed using an HPLC (ICS-500) with an Aminex HPX-87H column (Bio-Rad, Hercules, CA). The concentration of HMF standard (Sigma) ranged from 0.1 to 4.0 g/L, while the furfural standard concentration ranged from 0.1 to 2.0 g/L. The 0.45-um syringe filter (Chromatographic Specialties, Brockville, Canada) was used to filter all the standards and samples. 5 mM H_2_SO_4_ was employed as an eluent and the flow rate was 0.6 mL/min.

The concentration of total phenolics in the hydrolysates of the pretreated substrates was assessed by using the Folin–Ciocalteu reagent (Sigma), as proposed by Singleton and Rossi (1965). In brief, this method involves initially adding 250 μL of the Folin–Ciocalteu reagent into a 100-μL aliquot of the diluted sample. After 5 min, 750 μL of 20% (w/v) Na_2_CO_3_ was added to stop the reaction and the total volume is brought up to 5 mL using de-ionized purified water. The flasks were then placed on a magnetic stir plate with constant stirring at a temperature of 22 °C for 2 h. The absorbance of each reaction was measured using a UV spectrophotometer at 760 nm against a blank of de-ionized purified water. Vanillin was used as a calibration standard. The reported values are an average of duplicate measurements.

## Results and discussion

### Sulphite addition during steam pretreatment at the neutral/alkali conditions

Previous work (Zhong et al. 2019) had shown that lignin reactivity decreased after steam pretreatment, which could reduce the effectiveness of lignin sulphonation. In the work reported here, sulphite was added to the biomass during the steam pretreatment. A neutral or an alkaline pH was initially used with the expectation that the higher pH would limit acid-induced lignin condensation and the carbohydrate degradation that results in the formation of potential fermentation and hydrolysis inhibitors such as furans (Jönsson and Martín 2016). Previous work had successfully added alkaline sulphite to hardwoods with the goal of producing pulps for papermaking, which typically require fibers of maximum length and strength (Kokta and Ahmed 1998). Another work had indicated that an alkaline sulphite treatment resulted in sulphonation of the lignin, which consequently enhanced enzymatic hydrolysis while preserving the components of carbohydrate on biomass in the water-insoluble fraction (Chandra et al. 2016). However, the neutral and alkaline sulphite treatments either required multiple stages of steam pretreatment or a subsequent mechanical refining stage in order to obtain homogenous size reduction and fiber separation after the initial alkaline sulphite steam pretreatment process (Chandra et al. 2016; Kokta and Ahmed 1998). As related work had shown that homogeneous size reduction resulted in substrates which were more readily hydrolyzed when using steam-pretreated softwoods (Cullis et al. 2004), several steam pretreatment conditions and sulphite loadings were initially used over a range of concentration, times and pH (Table 1), to try to determine optimum conditions for carbohydrate recovery and cellulose hydrolysis.

**Table 1:** The acid group content, final pH and the enzymatic hydrolysis yield of the two-stage alkaline sulphite steam-pretreated substrates

Chemical loading	Condition	Acid group (µ mol/g substrates)	pH	Cellulose hydrolysis to glucose (%)
4% SO_2_	One stage—200 °C 5 min	–	1.7	81
16% Na_2_SO_3_	Stage 1—160 °C 10 min Stage 2—210 °C 10 min	96	4.9	30
16% Na_2_SO_3_	Stage 1—160 °C 20 min Stage 2—210 °C 10 min	110	4.6	36
16% Na_2_SO_3_	Stage 1—160 °C 20 min Stage 2—220 °C 10 min	105	4.1	35
16% Na_2_SO_3_	Stage 1—160 °C 20 min Stage 2—230 °C 10 min	101	3.8	38

Each steam pretreatment used a sulphite loading of 16% (based on dry biomass) at the indicated temperature and residence time. Enzymatic hydrolysis was conducted at a solids loading of 5% for 48 h using a cellulase loading of 35 mg protein per g cellulose

As a previous work had indicated that Na_2_SO_3_ impregnation prior to sulphonation was beneficial when applied to poplar/hardwood (Chandra et al. 2016), before steam pretreatment the lodgepole pine chips were soaked overnight in a Na_2_SO_3_ solution. However, it was shown that by using the neutral/alkaline sulphite in a single steam pretreatment stage, it did not result in substrates that underwent a homogenous size reduction (Additional file 1: Fig. S1A). As previous work using hardwoods (Chandra et al. 2016) had shown that two-stage alkaline sulphonation enhanced cellulose hydrolysis, the initial sulphonation was carried out at 160 °C while a subsequent, higher temperature (210–230 °C) was used to enhance fiber separation/size reduction and increase cellulose hydrolysis (Cullis et al. 2004). More acid groups were incorporated onto the softwood substrate after the two-stage pretreatment when 16% sulphite/160 °C was used in the initial pretreatment and a temperature of 200 °C used for the second steam pretreatment (Table 1). Although this enriched the acid groups content from 96 to 110 μ mol per gram dry substrates, similar to what had been observed previously, this two-stage pretreatment resulted in only limited fiber separation and size reduction and poor (38%) cellulose hydrolysis. It was apparent that the ease of hydrolysis of these two-stage sodium sulphite-pretreated softwood substrates was much lower than that of the single-stage SO_2_ steam-pretreated “control” substrate (81.5% hydrolysis) (Table 1). However, as the pH of the substrate after the two-stage pretreatment which incorporated sulphite ranged from pH 4 to 5 (Table 1) and increasing the pH has been shown to enhance sulphonation and fiber separation (Kokta and Ahmed 1998), in subsequent work, sodium carbonate was added with sodium sulphite to try to increase the alkalinity of the reaction.

### The effect of sodium carbonate addition for alkali sulphite steam pretreatment

**Fig. 1:**
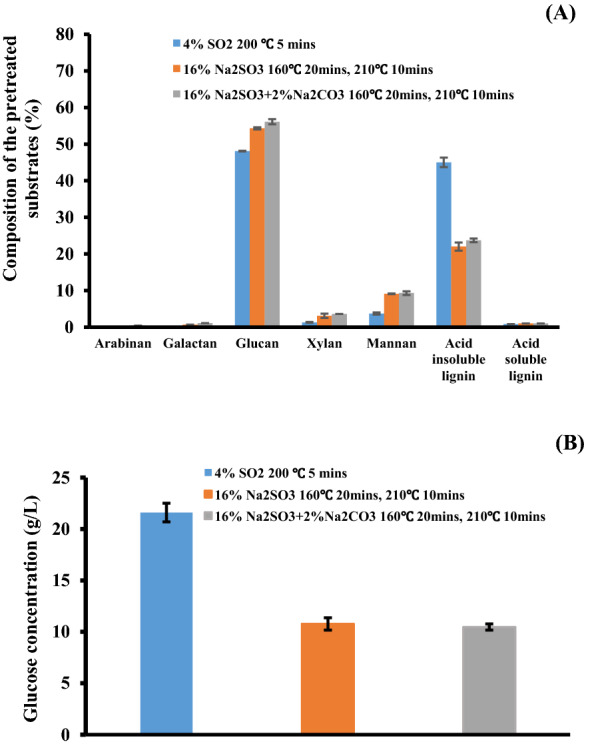
**A** Sugar and lignin composition of the substrates generated from the two-stage neutral/alkaline sulphite steam pretreatment of lodgepole pine with and without the addition of sodium carbonate. **B** The enzymatic hydrolysis yields of the washed substrates derived from two-stage alkaline sulphite steam pretreatment of lodgepole pine. Enzymatic hydrolysis was conducted at a solids loading of 5% for 48 h using a cellulose loading of 35 mg protein per g cellulose

As anticipated, the addition of 2% Na_2_CO_3_ to the 16% Na_2_SO_3_ during the steam pretreatment increased the sulphonation of the substrate. This increased the acid groups from 110 to 140 μ mol acid groups per gram substrates while enhancing carbohydrate retention in the water-insoluble fraction (Fig. 1A). The enhanced sulphonation and carbohydrate retention was likely a result of the higher pH environment (pH 4.6 vs. pH 6.4) provided by the added Na_2_CO_3_. However, despite the increased sulphonation, the particle size of the resulting substrates proved to be quite heterogeneous with relatively large particles still present (Additional file 1: Fig. S1B). Consequently, it is likely that the heterogeneous particle size of the substrate also resulted in much poorer cellulose hydrolysis, as the hydrolysis of the substrates treated with 16% Na_2_SO_3_ (glucose concentration 10.8 g/L, hydrolysis yield 36%) and 16% Na_2_SO_3_ + 2% Na_2_CO_3_ (glucose concentration 10.5 g/L, hydrolysis yield 34%) resulted in far lower glucose yields and sugar concentrations when compared to the softwood chips steam pretreated using 4% SO_2_ (glucose concentration 21.6 g/L, hydrolysis yield 81.6%) (Fig. 1B).

**Fig. 2:**
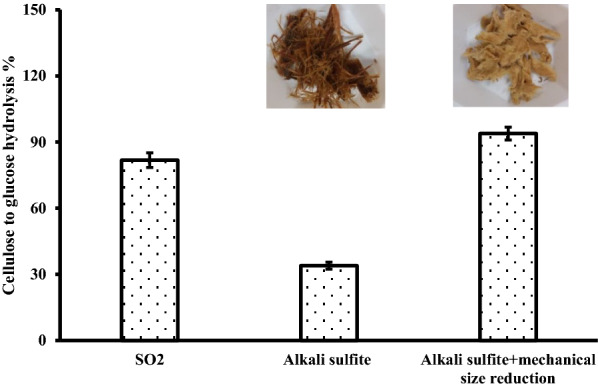
The influence of mechanical size reduction on the enzymatic hydrolysis of washed alkaline sulphite steam-pretreated lodgepole pine substrate. Enzymatic hydrolysis was conducted at solids loading of 5% for 48 h with a cellulase enzyme loading of 35 mg protein per g cellulose. The alkaline sulphite steam pretreatment was performed with 16% Na_2_SO_3_ and 2% Na_2_CO_3_ at 160 °C for 20 min followed by a second step of steam pretreatment at the temperature of 210 °C for 10 min. The mechanical size reduction was performed with a lab-scale grinder. The control sample was pretreated with 4% SO_2_ based on the dry biomass and steam pretreated at 200 °C for 5 min

As it was possible that the poor hydrolysis resulting from alkaline sulphite steam pretreatment was due to a lack of homogeneous size reduction, which previous work had shown was important (Chandra et al. 2008; Mooney et al. 1999; Cullis et al. 2003), the two-stage alkaline sulphite-pretreated sample was subjected to mechanical size reduction. This increased cellulose hydrolysis from 35 to 91%, indicating how important size reduction was in increasing cellulose hydrolysis (Fig. 2). The fiber length (0.859 mm) and fiber width (31.5 µm) of the alkaline sulphite-pretreated softwood was larger compared with that of the acidic steam-pretreated control (fiber length 0.41 mm, fiber width 27.3 µm) confirming enhanced fiber separation after alkaline sulphite treatment. This was anticipated as a similar sulphonation approach is typically utilized to enhance the fiber separation during chemithermomechanical pulping (CTMP) and alkaline sulphite steam explosion pulping (Kokta and Ahmed 1998). Thus, although steam pretreatment of softwoods using SO_2_ resulted in increased lignin condensation, it also resulted in effective size reduction. In contrast, although neutral/alkaline sulphite pretreatment enhanced lignin sulphonation, the sulphonated substrate was more heterogeneous and less readily hydrolyzed.

### Sulphite addition during the steam pretreatment at acidic conditions

Previous work using the Sulphite Pretreatment to overcome the Recalcitrance of Lignocellulose (SPORL) technique (Lan et al. 2013) had been shown to be relatively effective. Therefore, in the work reported here, an acidic bisulphite steam pretreatment was applied in two stages. Initially, the softwood chips were impregnated with a mixture of 10% bisulphite and 2% sulphuric acid and steam pretreated at either 180 °C for 20 min or 160 °C for 40 min. After this initial pretreatment, the temperature was subsequently raised to 200 °C followed by explosive decompression to try to emulate the mechanical refining step utilized in the SPORL process (Lan et al. 2013; Zhou et al. 2013). However, as indicated in Additional file 1: Fig. S2A it was apparent that insufficient size reduction occurred, resulting in poor (40%) cellulose hydrolysis. To try to resolve this issue, a single-stage acidic bisulphite treatment was used at a temperature of 210 °C. The cellulose and hemicellulose recovery were 92% and 72%, respectively, at the pretreatment condition of “4% SO_2_ 200 ℃ 5 min”, while their recovery reached to 100% and 88%, respectively, at the pretreatment condition of “8% NaHSO_3_ + 2% H_2_SO_4_, 210 ℃ 10 min”. At a solids loading of 25% (w/v), which was equivalent to a cellulose content of 103.3 g/L glucose, more than 58% of the cellulose could be hydrolzsed. This was slightly better than the yields obtained after hydrolysis of the SO_2_-catalyzed steam-pretreated softwood (Table 2).

**Table 2:** The sugar, lignin, acid group and potential inhibitor content as well as fiber length/width and cellulose hydrolysis of water soluble/insoluble fractions of SO_2_-catalyzed acid bisulphite steam-pretreated substrates

Samples	Water insoluble^a^										Water soluble^b^						Water-soluble inhibitors				pH	Enzymatic hydrolysis^c^
	Ara (%)	Gal (%)	Glu (%)	Xyl (%)	Man (%)	AISL (%)	Lignin removal (%)	Acid groups (μ mol/g substrates)	Fiber length (mm)	Fiber width (um)	Glu M (g/L)	Glu O (g/L)	Xyl M (g/L)	Xyl O (g/L)	Man M (g/L)	Man O (g/L)	Acetic acid (g/L)	Furfural (g/L)	HMF (g/L)	Total phenolics (g/L)		Glucan hydrolysis yield (%)
4% SO_2_ 200 ℃ 5 min	0	0.1	48.0	0.1	0.3	49.0	0.1	15.0	1.3	31.9	28.2	7.1	11.2	2.2	17.7	8.9	8.2	3.2	1.6	4.7	1.7	54.5
8% NaHSO_3_ + 2%H_2_SO_4_, 210 ℃ 10 min	0.1	0.2	62.1	1.4	2.2	32.5	33.0	88.0	0.4	27.3	0.6	3.3	1.5	0.6	2.5	4.8	0.9	0.3	0.2	7.6	3.6	58.1

The sugars, pH, and fermentation inhibitors present in the water-soluble fraction of acid bisulphite steam-pretreated lodgepole pine substrates based on 25% solid loading whole slurry

Enzymatic hydrolysis yields of single-stage acid bisulphite steam-pretreated lodgepole pine whole slurries. The hydrolysis was performed at a 25% solid loading for 48 h using a cellulase enzyme loading of 40 mg protein/g cellulose

^a^Water insoluble: Ara—arabinan, Gal—galactan, Glu—glucan, Xyl—xylan, Man—mannan, AISL—acid-insoluble lignin

^b^Water soluble: Glu—glucose, Xyl—xylose, Man—mannose, M—monomer, O—oligomer

^c^Enzymatic hydrolysis was conducted at 25% solids loading with a Ctec-3 enzyme loading of 40 mg protein/g glucan for 48 h

As indicated in Table 2 and Additional file 1: Fig. S2B, the single-stage sulphite treatment resulted in the good fiber separation and relatively long fibers with the incorporation of 88 μ mol/g of acid groups onto the pretreated substrate as compared to the 15 µ mol of acid groups presented on the 4% SO_2_ steam-pretreated substrate. The increased sulphonation also enhanced the separation and retention of larger, intact fibers, as the acid bisulphite treated substrate had an average fiber length of 1.3 mm as compared to 0.4 mm for the SO_2_ steam-pretreated substrates (Table 2). Enhanced sulphonation also resulted in its partial lignin solubilization, as 33% delignification was detected after pretreatment (Table 2). Although it was shown longer fibers are detrimental to the liquefaction step of cellulose hydrolysis (Zwan et al. 2017), good hydrolysis with 58% yield was obtained and it was slightly higher than the 54% for SO_2_ steam-pretreated substrate (Table 2).

### Fermentability of the acidic sulphite steam-pretreated whole slurry

**Fig. 3:**
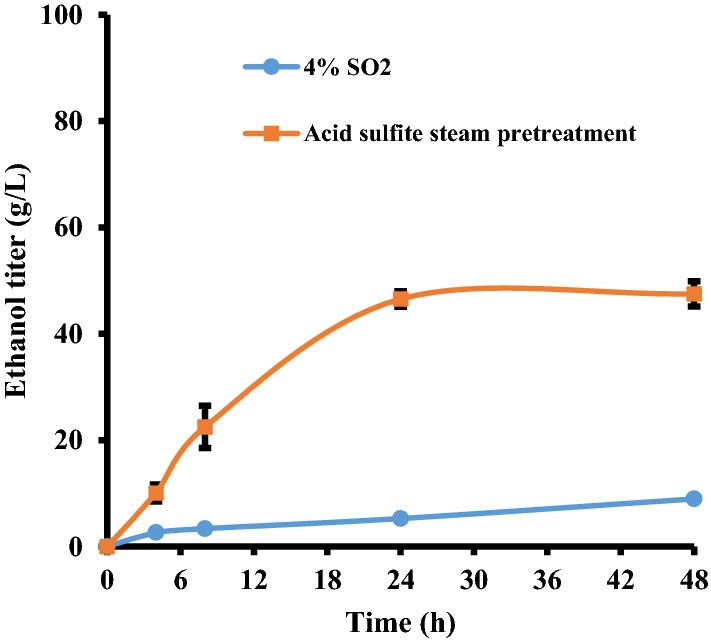
Ethanol production profile of *S. cerevisiae* strain T_2_ fermentation of whole slurry hydrolysate after one-stage acid bisulphite or SO_2_-catalyzed steam pretreatment of lodgepole pine. The one-stage sulphite steam pretreatment was performed by using 8% NaHSO_3_ and 2% H_2_SO_4_ at 210 °C for 10 min. Enzymatic hydrolysis of the sulphite steam-pretreated substrates was conducted at a solids loading of 25% for 48 h with a cellulase loading of 40 mg protein per g cellulose. The yeast concentrations (cell optical density: OD) were 6.5

As well as enhancing cellulose hydrolysis, we also hoped that the sulphite treatment would help detoxify the steam-pretreated softwood. As indicated in Table 2, the acid bisulphite treatment decreased the amount of furans and acetic acid from 4.8 to 0.45 g/L and 8.2 to 0.91 g/L, respectively, while the phenolics increased from 4.7 to 7.6 g/L with the increase in phenolic compounds likely due to the sulphite-induced fragmentation of the lignin (Sjöström 1993). However, it was possible that the inhibitory nature of the phenolics might have changed, as previous work had shown how sulphonation resulted in reduced phenol toxicity (Jönsson et al. 2013) while the same treatment also reduced furan toxicity (Alriksson et al. 2011). To try to assess the extent of cellulose hydrolysis and ease of fermentation of the released sugars, acid bisulphite steam-pretreated lodgepole pine whole slurries (25% solid loading) which had been hydrolyzed and fermented produced 46.6 g/L of ethanol after just 24 h (Fig. 3). This was significantly better than the ethanol yields obtained from the SO_2_-catalyzed steam-pretreated softwood (Fig. 3), which was poorly fermented, likely due to the presence of inhibitors (Zhong et al. 2019). In summary, acid bisulphite treatment applied during the steam pretreatment process increased the ease of enzyme-mediated hydrolysis of the cellulose component while enhancing the fermentability of the cellulose and hemicellulose-derived sugars present in high-consistency softwood substrates.

## Conclusions

The lignin in the softwood chips could be readily sulphonated during the steam pretreatment. Although the use of alkaline conditions during sulphite treatment maximized lignin sulphonation, it did not result in a substantial increase in the enzymatic hydrolysis of cellulose. This was likely due to insufficient size reduction and the heterogeneous nature of the pretreated chips. In contrast, an acidic sulphite approach, which used a combination of sulphuric acid and sodium bisulphite, resulted in a more homogeneous size reduction and fiber separation, likely due to enhanced lignin solubilizing. It was apparent that acid sulphite steam pretreatment improved both the enzyme-mediated hydrolysis of the carbohydrates and the fermentability of the liberated sugars when using high substrate concentrations.

### Supplementary Information

**Additional file 1: Figure S1.** **A** Softwood chips after one-stage alkaline sulphite steam pretreatment with 16% Na_2_SO_3_ loading (per gram of the dry biomass) at 160 ℃ for 70 min. **B** Softwood chips after two-stage alkaline sulphite steam pretreatment using a 16% Na_2_SO_3_ loading (per gram of the dry biomass) and a range of carbonate loadings (**B-1**: 2%, **B-2**: 4% and **B-3**: 6%) using steam pretreatment conditions of 160 ℃, 20 min for the first stage and 210 ℃, 10 min for the second stage. **Figure S2.** **A** Single and two-stage acid bisulphite steam-pretreated lodgepole pine substrates. The single-stage acid-sulphite steam pretreatment was performed using 8% NaHSO_3_ and 2% H_2_SO_4_ at 210 °C for 10 min. The two-stage acid bisulphite was performed using 8% NaHSO_3_ and 2% H_2_SO_4_ at 160 °C for 20 min followed by a second steam pretreatment stage at 210 °C for 10 min. **B** FE-SEM images of the one-stage acid bisulphite (8% NaHSO_3_ and 2% H_2_SO_4_ at 210 °C for 10 min) and 4% SO_2_-catalyzed acid steam-pretreated lodgepole pine substrates.

## Data Availability

The data generated and/or analyzed during this study are available from the corresponding author on reasonable request.

## Abbreviations

WIF: Water-insoluble fraction
WSF: Water-soluble fraction
FQA: Fiber quality analyzer
SEM: Scanning electron microscopy
FID: Flam ionization detector
HMF: 5-Hydroxymethyl furfural
CTMP: Chemithermomechanical pulping
SPORL: Sulphite Pretreatment to Overcome the Recalcitrance of Lignocellulose
