# Supplementary material for: Sulphite addition during steam pretreatment enhanced both enzyme-mediated cellulose hydrolysis and ethanol production
Source: Bioresour Bioprocess. 2022 Jun 29;9(1):71. doi: 10.1186/s40643-022-00556-w (PMC10991184; doi:10.1186/s40643-022-00556-w)
Supplement: Supplementary file 1 — Additional file 1: Figure S1. A Softwood chips after one-stage alkaline sulphite steam pretreatment with 16% Na2SO3 loading (per gram of the dry biomass) at 160 ℃ for 70 min. B Softwood chips after two-stage alkaline sulphite steam pretreatment using a 16% Na2SO3 loading (per gram of the dry biomass) and a range of carbonate loadings (B-1: 2%, B-2: 4% and B-3: 6%) using steam pretreatment conditions of 160 ℃, 20 min for the first stage and 210 ℃, 10 min for the second stage. Figure S2. A Single and two-stage acid bisulphite steam-pretreated lodgepole pine substrates. The single-stage acid-sulphite steam pretreatment was performed using 8% NaHSO3 and 2% H2SO4 at 210 °C for 10 min. The two-stage acid bisulphite was performed using 8% NaHSO3 and 2% H2SO4 at 160 °C for 20 min followed by a second steam pretreatment stage at 210 °C for 10 min. B FE-SEM images of the one-stage acid bisulphite (8% NaHSO3 and 2% H2SO4 at 210 °C for 10 min) and 4% SO2-catalyzed acid steam-pretreated lodgepole pine substrates. [file 40643_2022_556_MOESM1_ESM.docx]

**
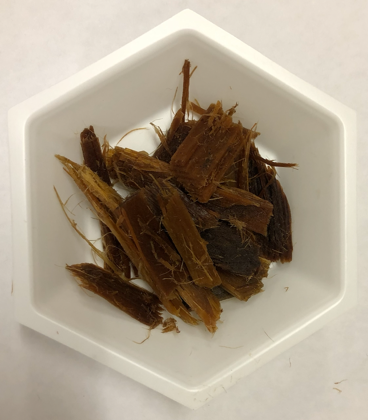
**

(A)


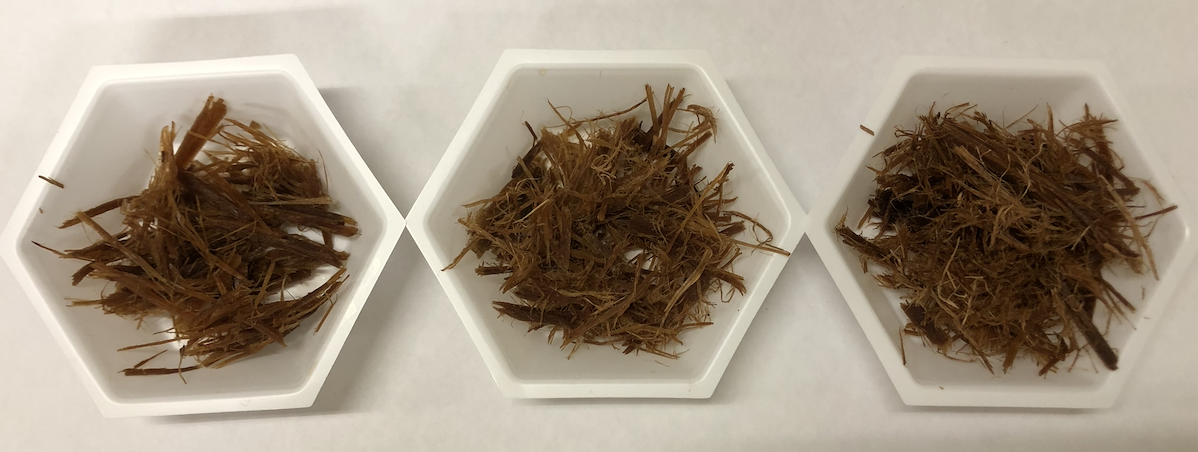


(B-1)

(B-2)

(B-3)

***S* 1.** (A) Softwood chips after one stage alkaline sulphite steam pretreatment with 16 % Na_2_SO_3_ loading (per gram of the dry biomass) at 160 ℃ for 70 min. (B) Softwood chips after two-stage alkaline sulphite steam pretreatment using a 16 % Na_2_SO_3_ loading (per gram of the dry biomass) and a range of carbonate loadings (B-1: 2%, B-2: 4% and B-3: 6%) using steam pretreatment conditions of 160 ℃, 20 min for the first stage and 210 ℃, 10 min for the second stage.

**(A)**


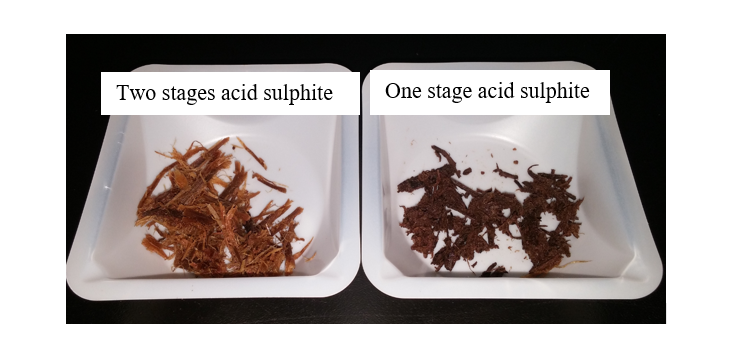


**(B)**

| 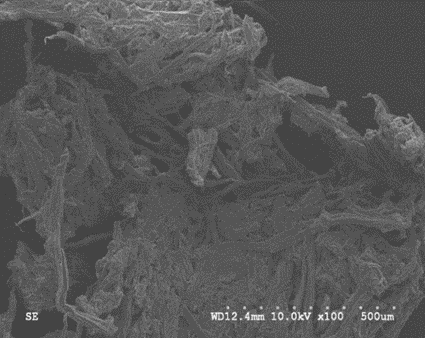 | 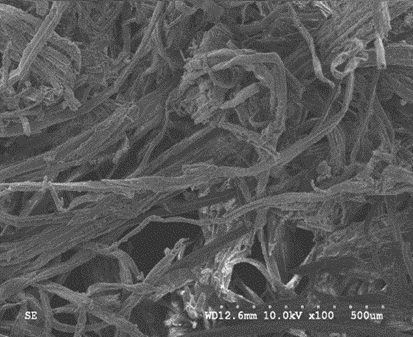 |
| --- | --- |
| 4% SO_2_ acid steam pretreated substrate | One stage acid bisulphite steam pretreated substrate |

***S* 2.** (A) Single and two-stage acid bisulphite steam pretreated lodgepole pine substrates. The single stage acid-sulphite steam pretreatment was performed using 8% NaHSO_3_ and 2% H_2_SO_4_ at 210 °C for 10 min. The two-stage acid bisulphite was performed using 8% NaHSO_3_ and 2% H_2_SO_4_ at 160 °C for 20 min followed by a second steam pretreatment stage at 210 °C for 10 min.

(B) FE-SEM images of the one stage acid bisulphite (8% NaHSO_3_ and 2% H_2_SO_4_ at 210 °C for 10 min) and 4% SO_2_ catalyzed acid steam pretreated lodgepole pine substrates.
